# The Treatment of Syphilis

**Published:** 1883-05

**Authors:** 


FROM FOREIGN JOURNALS.

*The Treatment of Syphilis.*—More than forty years ago, I practiced medicine in Montgomery county, Alabama, near the Creek nation of Indians. Syphilis was then very prevalent among them, and their medicine men had the reputation of speedily curing it. Their remedies were, of course, decoctions of native herbs. It was generally known that queen's delight (*Stillingia sylvatica*) was one of their principal agents. I had supposed that, when this tribe were removed west of the Mississippi in 1837, their secret of curing syphilis had gone with them; but, when I was in Alabama last year, I learned from my brother-in-law, Dr. B. Rush Jones, of Montgomery, the following facts touching this question.

There were, he said, seven or eight years before our civil war, several obstinate cases of secondary syphilis in and around Montgomery, which resisted the usual remedies in the hands of our best physicians. They went the round of the doctors, and could not be cured. At last one of these was advised to consult a colored man, Lawson, belonging to Mr. N. D. Barnett, a cotton planter residing in Montgomery county. In a state of despair, he went to see Lawson, put himself under his treatment, and in a few weeks he was perfectly cured. He returned to town rejoicing at his recovery, and soon others of his fellow-sufferers followed his example, went to consult the colored man, Lawson, and were likewise cured. These cures by an obscure negro man, a slave, when the highest representatives of science had failed, were much spoken of in both town and country, and attracted the attention of Dr. Geo. W. McDade, a very intelligent and accomplished physician, whom I have known since his early boyhood. Dr. McDade feeling the greatest interest in the subject, went to see Lawson, who had made these marvelous cures, and obtained from him the formula he had been using so successfully.

Soon after this, Dr. McDade happened to meet Dr. Jas. Freeny, who gave him the following history of the so-called Indian method of treating syphilis. Horace King, a mulatto slave, resided among the Creek Indians for several years before they were removed west of the Mississippi river (1837), and had learned from them their method of treating syphilis. While Horace was engaged in building a bridge at Tallahassee, about twenty-five miles from Montgomery, in 1852, he heard that there were many cases of syphilis on Mr. Gipson's plantation near by, and that Drs. Freeny and Banks were the attending physicians; and he called on Dr. Freeny, and told him that he had learned a method of treating syphilis from the Creek Indians, which was universally successful, and that he would like to show it to him. And for this purpose he proposed to take the worst cases on the Gipson plantation for the experiment. Drs. Freeny and Banks selected a certain number of very bad cases, and turned them over to Horace; and they watched from day to day his method, while they continued their own plan with the other cases.

Horace's selected bad cases recovered more rapidly than Dr. Freeny's milder ones, and then Dr. Freeny adopted the Indian method in the other cases on the Gipson plantation, and has not pursued any other plan since.

So thoroughly convinced was Dr. Freeny of the superiority of the Indian remedy, that he wrote to Dr. Warren Stone, Professor of Surgery in the University of Lousiana, urging him to give it a trial in the wards of the great Charity Hospital of that city.

Dr. Freeny failed to enlist the interest of Professor Warren Stone in the matter, and he made no further effort to bring it before the profession, except by speaking of it to his brethren in his immediate neighborhood.

After Horace's success on the plantation of Mr. Gipson, and the adoption of his method by the two well known physicians Drs. Freeny and Banks, Mr. Nicholas D. Barnett, a large cotton-planter, sdnt his servant Lawson, a very intelligent man (before alluded to), to Horace King to learn his remedies, and the method of preparing and using them. Horace readily imparted the desired information, and Lawson returned home, and put the treatment to the test among the negroes on his master's plantation. It was as successful in the hands of Lawson as it had been in those of Horace King.

After a while, other planters in Mr. Barnett's neighborhood followed his example, and set apart confidential servants to take charge of syphilitic cases, and treat them with the Indian decoction. And thus several adjoining plantations had each its negro doctor, all using the same method with equal success.

This was in a rich section of Montgomery County, where there were many large cotton-plantations in juxtaposition; some of one thousand acres, some of two thousand and more, having from one to two or three hundred slaves on each, while there were others of less size with fewer slaves.

On some plantations—notably, on Mr. Barnett's—the syphilitic cases, male and female, were sent to a hospital specially set apart for the purpose, and there quarantined till they were cured. They were, during the period of treatment, wholly cut off from all communication with the other negroes on the plantation. This was in the time of slavery, when the intelligent and humane master had the right to protect his people against infectious diseases of all sorts. Syphilis was thus controlled, and small-pox effectually stamped out, because the sanitary state of the plantation was intrusted to medical men of the highest intelligence, who were authorised by the master to do all that was necessary for the health of the community.

Dr. McDade says: "It is very remarkable how few cases of secondary syphilis, scrofula, and consumption, existed in those days among the slaves, compared with what we now find. The two latter were then almost unknown among the negroes; but since emancipation they are very common."

"Is secondary syphilis the parent of scrofula and consumption? Certainly, these were rarely seen among the negroes while in slavery; whereas they are now encountered every day. Secondary syphilis was then less frequent among them than now, because their masters took every precaution for their early treatment and cure. But now the negro is free to contract this loathsome disease, and to scatter it as he may. You may ask, Why are they not treated? I answer, Many never apply for treatment; and, when they do, they often disappear before they are cured. And many of them are too poor or too improvident to apply for treatment. Physicians, always the conservators of the public health, never here refuse to treat a case of syphilis because the subject of it is a freedman, poor and improvident."

Professor Samuel D. Gross read an exhaustive paper on the connection between syphilis and scrofula and consumption, before the American Medical Association in 1875, advocating the view that the two latter were the offspring of syphilis, and it would now appear that the history of these in the negro, in slavery and in freedom, goes far to establish the correctness of the views so forcibly set forth by my distinguished countrymen.

Dr. McDade says, "that the remedies used by Lawson on Mr. Barnett's plantation, were the same as those used by Horace King. They consisted of ten or a dozen indigenous roots, a handful of each, with a certain quantity of salt, alum, and iron slugs put into three gallons of water, and boiled down to one gallon. Of this the patient took a half pint three times a day. There was also a decoction of roots for washing the syphilitic sores. After obtaining these prescriptions, it was a long time before I made any trial of their virtues. I was deterred by the fact that it would be difficult for any patient to drink and retain half a pint, three times a day, of such a vile decoction. The horrors of syphilis could alone inspire a man with courage to take it. However, I saw that those who did were invariably relieved, whether in the first, second, or third stage of the disease."

"Instead of adopting the so-called Indian remedy as I found it, I began by eliminating the alum, salt, iron nails, and slugs, and all the roots and herbs that I knew must be absolutely inert. I selected the few among them known to possess medicinal properties; and, instead of making a decoction as had been done before, and which had to be made in large quantities every day or two, I had them prepared in the form of fluid extracts, which places the remedy on a scientific basis, and insures uniformity of action. The following is the formula that I and my medical friends have been using for many years."

"Fluid extract of smilax sarsaparilla, fluid extract of stillingia sylvatica (queen's delight), fluid extract of lappa minor (burdock), fluid extract of phytolacca decandra (poke root), aa ℥ij, tincture of xanthoxylum carolinianum (prickly ash), ℥j. Take a teaspoonful in water three times a day before meals, and gradually increase to tablespoonful doses.

"In making the fluid extracts, there is some risk of getting a remedy less efficient than the original Indian decoction, because the manufacturer may use roots that have been kept too long, and lost some of their active principles, while the decoction used on the plantations was always made of fresh roots just gathered from the woods.

In making the fluid extracts, we should therefore be careful to have them made from roots recently gathered." While Dr. McDade makes fluid extracts of four of his ingredients, he makes a tincture of the fifth. I do not understand why he did not order a fluid extract of that also. I simply give the prescription as it was given to me by Dr. McDade and Dr. Rush Jones.

Stillingia sylvatica has long been used in the Southern States as an antisyphilitic remedy by both the profession and the laity. Professor Thomas Y. Simons, of Charleston, was the first to call our attention to it (*American Medical Recorder*, 1828). His favorable report was subsequently confirmed by Professor Henry R. Frost, of Charleston, and by Dr. A. Lopez, of Mobile, Alabama, (New Orleans *Med. and Surg. Journal*, 1846). Dr. Frost thinks the active principle of the stillingia is somewhat volatile, and says that the root loses much of its activity when kept long. I know that the odor of the recent root is much stronger than the dried. I presume the stillingia sylvatica and the smilax sarsaparilla are the efficient agents in McDade's compound fluid extract. Dr. McDade says: "I could detail many cases illustrating the wonderful antisyphilitic powers of this remedy; but I will give you only two. 1. A young negress contracted sphilis from her husband, who resided on a neighboring plantation, and visited his wife generally about twice a week. This was long before the war (1861). They were both treated by the late Dr. Afred McDonald, and they were apparently cured. But they had several children subsequently, and in rapid succession, all of whom died of syphilis soon after birth. The husband and wife were then treated by the Indian decoction, and were permanently cured, as shown by the fact that they had several healthy children afterwards at full term, who grew to manhood and to womanhood. None of them ever showed any signs of syphilis, nor have any of their children. Those of them who have died, died of other diseases of a climatic character."

"2. A negro girl, twenty years old, belonging to Mr. Cobb, had syphilitic iritis. This case had resisted all treatment by the best physicians of the country. She was nearly blind. She was taken in charge by Mr. Barnett's colored man, Lawson who gave her the Indian remedy, and she was perfectly and permanently cured, as she never afterwards showed any symptom of the disease. These cases occurred more than twenty-five years ago; and have been under my observation ever since, so you will see that the cures are permanent."

"Mr. Barnett has pursued the same method on his plantation since emancipation that he did during slavery. His man Lawson uses the same compound decoction now that he did in olden times, and cures many cases every year on Mr. Barnett's plantation, and on those adjoining."

Dr. McDade has used his compound as an alterative with great success in scrofula, and he thinks it would be worth trying in some forms of cancer.

Dr. Rush Jones, residing in the city of Montgomery, has a larger field of observation than Dr. McDade, residing in the country, and has really had a larger experience with McDade's antisyphilitic fluid extract than any one else; and he speaks most favorably of it. He has been treating syphilis for more than forty years, and he says he now has but little dread of undertaking the worst cases, since he has adopted the use of McDade's formula. He repudiates mercury and the iodide of potassium entirely, and says they are unnecessary when McDade's formula is used.

Dr. Rush Jones says: "It is a remarkable fact that I do not see more than one case of syphilis in woman to fifty cases in the male. I have inquired of a number of physicians in regard to this fact, and their experience coincides with mine. How can this be accounted for?"

I am not familiar with the literature of syphilis, and do not know if the fact alluded to by Dr. Rush Jones has been observed in other parts of the world. If so, it seems to me to have an important bearing on the practical application of the Contagious Diseases Acts. And so would the complete history of the working of the quarantine and isolation of infected negroes on the several cotton plantations in Montgomery county, Alabama, during the time of slavery and since emancipation, if we could obtain minute and reliable reports on the subjects

I am no authority on the subject of syphilis; and, if any apology were necessary for this communication, it is this:

I was at the meeting of the London Medical Society on November 26th last, and heard the discussion on the papers of Dr. Drysdale and Dr. Routh on syphilis. From this, it appeared that we now differ as widely on the subject of its treatment, as we did fifty years ago. And this gave me the idea of writing to Dr. Rush Jones and Dr. McDade for the facts which I now lay before the profession.

I have known Dr. Rush Jones all my life, and I have known Dr. McDade, and Dr. Freeny, and Mr. Barnett, for more than forty years, and have perfect confidence in any statement they might make, or I would never have said a word on this subject. I think great credit is due to Dr. Freeny and Dr. Banks for giving the colored man Horace King an opportunity to demonstrate the value of the Indian decoction in the treatment of syphilis on the plantation of Mr. Gipson. For its success there brought it, with their endorsement, prominently before the community, and extended its use to the plantations of Mr. Barnett and his neighbors.

Too much credit cannot be given to Dr. McDade for investigating the subject, and giving us a formula at once scientific and efficient; for it has proven efficient in the hands of Dr. Rush Jones, Dr. McDade, and many other physicians who have been using it for several years past.

I should be pleased to see the name of McDade used by the profession hereafter to designate the formula and the method of treatment herein set forth. The remedy will doubtless be extensively used, at leastfor awhile; and I sincerely hope it may prove as efficient here as it has in the hands of my friends in Montgomery, Alabama.—*J. Marion Sims, M.D., in British Medical Journal.*

